# Napping and heart rate variability in elite athletes

**DOI:** 10.5114/biolsport.2024.132983

**Published:** 2024-02-07

**Authors:** Maher Souabni, Mehdi J Souabni, Sami Hidouri, Achraf Ammar, Mohamed Younes, Omar Hammouda, Tarak Driss

**Affiliations:** 1 Interdisciplinary Laboratory in Neurosciences, Physiology and Psychology: Physical Activity, Health and Learning (LINP2), UPL, UFR STAPS (Faculty of Sport Sciences), Paris Nanterre University, Nanterre, France; 2 Research Laboratory, Molecular Bases of Human Pathology, LR19ES13, Faculty of Medicine, University of Sfax, Sfax, Tunisia; 3 Department of Training and Movement Science, Institute of Sport Science, Johannes Gutenberg University Mainz, Mainz, Germany; 4 High Institute of Sport and Physical Education of Sfax, University of Sfax, Sfax, Tunisia; 5 COSMED France, Rue Henri Malartre – Zac de Sacuny, 69530 Brignais, France

**Keywords:** Heart rate, Sleep, Autonomic Nervous System, Athletes, Basketball

## Abstract

Sleep and autonomic nervous system (ANS) influence each other in a bidirectional fashion. Importantly, it has been proposed that sleep has a beneficial regulatory influence over cardiovascular activity, which is mostly controlled by autonomic regulation through the activity of sympathetic and parasympathetic pathways of the ANS. A well-established method to non-invasively assess cardiac autonomic activity is heart rate variability (HRV) analysis. We aimed to investigate the effect of a 40-min nap opportunity on HRV. Twelve professional basketball players randomly accomplished two conditions: 40-min nap (NAP) and control (CON). Nocturnal sleep and naps were monitored by actigraphic recording and sleep diaries. Total sleep time (TST), time in bed (TIB), sleep efficiency (SE), sleep onset latency (SOL), and wake after sleep onset (WASO) were analyzed. HRV was analyzed in 5-min segments during quiet wake before and after each condition with controlled breathing. Were analysed high (HF) and low frequency (LF) bands, the standard deviation of NN interval (SDNN), HRV index and stress index (SI). Wellness Hooper index and Epworth Sleepiness Scale (ESS) were assessed before and after both conditions. There was no significant difference in TIB, TST, SE, WASO, and VAS between NAP and CON. A significant increase in SDNN, HRV index, and LF and a significant decrease in HF, SI, ESS, and Hooper’s stress and fatigue scores were observed from pre- to post-nap. In conclusion, napping reduces sleepiness, stress and fatigue, and might provide an advantage by preparing the body for a much-required sympathetic comeback following peaceful rest.

## INTRODUCTION

Sleep has been widely recognized as the most effective recovery strategy available to athletes [1, 2]. In the last decade, a growing body of evidence has proposed napping as a safe and non-invasive intervention to supplement night-time sleep and improve physical and cognitive performances [3–6]. In our previous research, we reported a beneficial effect of napping on physical and cognitive performances in athletes [7] as well as in older adults [8]. A 40 min daytime nap opportunity improved physical outcomes of elite basketball players and was deemed successful as a strategy to overcome the deterioration in shooting performance caused by the fatigue induced during exhaustive gameplay situations [9]. In another study, we reported a beneficial effect of napping on technical and tactical performance, physiological responses, and perceived exertion during a real game situation among elite athletes [10]. In addition, napping was an effective strategy for reducing sleepiness, stress, fatigue, anxiety, anger, and oral temperature [9, 10]. However, the mechanisms underlying the beneficial effects of napping remain unclear. According to Gupta, “Siesta is still an enigma” [11].

Furthermore, it has been reported that sleep and the autonomic nervous system (ANS) influence each other in a bidirectional fashion (for review see Trinder et al. [12]). Changes in the ANS modulate sleep onset as well as the transition between the different stages during nocturnal sleep [12, 13] but also during daytime naps [14, 15]. Importantly, it has been proposed that sleep has a beneficial regulatory influence over cardiovascular activity, a reciprocated influence that likely reflects a functional aspect of sleep [12]. A well-established method to non-invasively assess cardiac autonomic activity is heart rate variability (HRV) analysis [14, 15]. The cardiovascular system is mostly controlled by autonomic regulation through the activity of sympathetic and parasympathetic pathways of the autonomic nervous system. Analysis of HRV permits insight into this control mechanism [16, 17].

Moreover, HRV represents a psychophysiological marker of mental and physical well-being [18–20]. Increased HRV reflects a healthy ANS that can respond to changing environmental circumstances [19, 21]. By contrast, decreased HRV is a marker of autonomic inflexibility [22] and has been linked to a very large number of physical [23, 24] and psychological [25, 26] diseases. Furthermore, several studies have shown that acute [27, 28] and chronic [29, 30] stresses lead to a decrease in HRV. In the area of sports, HRV has increasingly been used to examine training load and recovery state after training [31], to monitor changes in physical performance and individual adaptation to training [32], to assess ventilatory thresholds [33], etc…

Fundamentally, HRV consists of measuring the inter-beat time intervals between consecutive heartbeats and represents the variability of intervals between consecutive R-peaks (RR) on the QRS complex on the ECG [17]. HRV measures can be divided mainly into time domain measures, based on arithmetic calculations of RR intervals; and frequency domain measures, based on spectral analysis (see Malik et al. [34] for more information on methods of HRV analysis). Studies investigating the effect of daytime napping on HRV in healthy young individuals reported a reduction in cardiovascular output and a shift from sympathetic to parasympathetic (vagal) dominance from wakefulness into sleep [14, 35, 36]. Importantly, these circadian- and sleep-dependent shifts in the ANS are critical to the maintenance of autonomic balance between parasympathetic and sympathetic branches and are beneficial for health and cognition [37, 38]. Moreover, Suppiah et al. [39] reported that nap did not elicit any performance or physiological benefits as monitored by HRV among adolescents. Another study aimed to explore the relationship between HRV and napping duration and their impact on handball performance [40]. Although no significant difference was reported between the two nap opportunities (i.e., 20 and 60 min) on HRV, a significant correlation was showed only with long nap between HRV parameters and handball performance.

Given the pressing need to understand the role of napping on physiological and psychological health and the lack of studies investigating the effect of daytime sleep on HRV in athlete populations, the present study aimed to assess cardiac autonomic activity — through HRV analysis — before and following an afternoon nap in elite basketball players. We hypothesize that diurnal napping would (i) change the HRV profile of athletes following nap in order to prepare them for physical stress, and (ii) impact positively perceived variables including stress, sleepiness and fatigue.

## MATERIALS AND METHODS

### Participants

Twelve high-level professional male basketball players (26 ± 5 years; 193 ± 7 cm; 87 ± 11 kg; 13 ± 2 % body fat and 17 ± 6 years expertise) volunteered to participate in the study. They were fully informed about the study information, and written informed consent was obtained from each participant before the study. Athletes are classified as successful elite (eliteness’ mean score = 9.0 ± 1.2), based on Swann et al. [41]’ categories. Eight out of the twelve players have taken part in international tournaments and represented their country in international competitions. Seven of them have more than 8 years of experience at the highest level of competition. Players were part of the same team and they trained regularly 6–8 times per week and played 1–2 competitions per week since they signed a professional contract (*i.e.,* 8 ± 5 years). They were asked to stay away from tobacco, alcoholic, or caffeinated beverages. None was habitual napper or presented an extreme morning or extreme evening type (Morningness–Eveningness Questionnaire’s mean score = 56 ± 3.1) [42]. The protocol of the present study was approved by the local Institutional Review Board (CPP SUD N° 0339/2021) and carried out according to the guidelines of the Helsinki Declaration for human experimentation. The sample size was a priori calculated using the G*power software [43], as strongly recommended [44], and based on an earlier study with a similar paradigm [45]. Statistical analysis indicated a minimum required sample size of twelve participants.

### Procedure

To minimize the learning effects during the study, participants were familiarized with the experimenters, sleeping room, tests, and questionnaires during a familiarization session. In addition, to assess their maximal heart rate (HR_max_), players carried out a Yo-Yo Intermittent Recovery test level-1; which is considered a valid basketball-specific test for the assessment of aerobic fitness [46]. The test consisted of 20-m shuttle runs performed at increasing velocities with 10-s active recovery between runs until exhaustion.

In experimental sessions, each participant completed randomly two test sessions, 72 hours apart. All sessions were performed after a reference night. Athletes had a standardized morning in the laboratory. They subjectively rated their last night’s sleep, ate a standardized breakfast (8:00 h) then stayed awake doing passive activities (*e.g.,* watching television, reading). After eating an isocaloric lunch at 12:00 p.m., they were assigned to experience nap (NAP) and no-nap (CON) conditions. HRV was analyzed in 5-min segments during a quiet wake before and after each condition with controlled breathing in a supine position.

In NAP condition, participants entered the comfortably warm, fully dark, and quiet sleeping room at 12:50 h. After 10 min of acclimatization in bed, nap opportunity started at 13:00 h and lasted for 40 min. The visual analogue scale (VAS) was presented to participant following the nap opportunity to evaluate their subjective sleep quality. In the CON condition, participants spent the same amount of time seated in comfortable chairs watching television.

### Measured Variables

#### Actigraphy and sleep diaries

Participants wore GT3X activity monitors (Actigraph, Pensacola, FL, USA) on their non-dominant arms the night before each experimental day (from 18:00 h) and took them off after the napping opportunities (at 15:00 h). Sleep parameters (TST, time in bed (TIB), sleep efficiency (SE), sleep onset latency (SOL), and wake after sleep onset (WASO)) were derived and analyzed using Actilife 6 (version 6.13.7) software. Actigraphy is a non-invasive device and was evaluated as a valid tool to assess sleep and wake behavioursbehaviours compared to the gold standard polysomnography [47].

### Subjective sleep quality

The subjective sleep quality was evaluated using the visual analogueanalogue scale (VAS) [48]. The VAS is a 10-cm scale that shows “Very bad sleep quality” (left side) and “Very good sleep quality” (right side).

### Heart rate (HR)

Heart rate was assessed using HR monitor chest belts (Team System 2, Polar, Kempele, Finland) provided with internal memory and recorded at 1-second intervals. The HR beats were exported and analyzed using Excel software (Microsoft Corporation, Redmond, WA, USA). HR data were expressed as mean (HR_mean_), peak (HR_peak_), and percentage of each subject’s individual HR_max_ (%HR_max_).

### Heart rate variability (HRV) analysis

R-wave peaks were detected automatically by Kubios HRV Analysis Software 2.2 (Matlab, Kuopio, Finland), visually examined, and edited for artefacts [49]. The same software was employed to perform the HRV analysis of the R-wave series according to the Task Force of the European Society of Cardiology and the North American Society of Pacing and Electrophysiology guidelines [34]. Kubios HRV is a cutting-edge and simple-to-use freeware for coronary heart rate variability (HRV) analysis. It comprises an improvised QRS detection algorithm and tools for noise correction, trend removal, and analysis sample selection [50]. The two major components of the frequency domain, i.e., high frequency (HF, 0.15–0.4 Hz) and low frequency (LF, 0.04–0.15 Hz) bands, and for the time domain, the standard deviation of the NN interval (SDNN), an index of global variability, were analyzed. Moreover, HRV triangular index (HRV index) and stress index (SI) were also calculated. HRV index is a geometric measure that calculates the integral of the density of the RR interval histogram divided by its height and reflects the global HRV. A 5-min epoch is conventionally used to study the above-mentioned variables [51]. Normalized HRV values (LF nu, HF nu) were calculated from the raw values of either short-term frequency band (LF or HF) divided by the total spectral power (typically LF + HF), with the value of this expressed as a decimal [52]. Unlike raw power, normalized units allow direct comparison between frequency and autoregressive methods for calculating spectral power, between spectral power expressed as ms^2^ or bpm^2^, and between different algorithms for calculation.

### Hooper scale

This is a validated psychological self-reporting scale of sleep quality, fatigue, stress, and muscle pain. Parameters were measured separately using a 7-point subjective rating scale ranging from 1 “very, very low” to 7 “very, very high”. The total score indicates the athlete’s form state or readiness to train [53].

### Epworth Sleepiness Scale (ESS)

Daytime sleepiness was assessed using a scale of eight elements (*i.e.,* ESS). Participants assign a score of “0” to “3” for each situation where there is “no chance” for “0”, a low chance for “1”, “a moderate chance” for “2” and “a high chance” for “3” to fall asleep. The score obtained from the scale ranges from “0” to “24” [54].

### Statistical analysis

Analyses were performed using Excel (Microsoft Office, v.2016) and SPSS Statistics (IBM, v.23) software. All data were expressed as means ± standard error of the mean (SEM). The Shapiro-Wilk W-test revealed that the normalized units of LF (LF nu) and HF (HF nu), SDNN, HRV index, ESS and Hooper’s fatigue and total score were normally distributed. Analysis was performed using a two-way repeated measures ANOVA [2 conditions (CON and NAP) x2 times (pre and post)] for HR_mean_, HR_peak_, LF nu, HF nu, SDNN, HRV index, ESS, Hooper’s fatigue, and the total score. ANOVA effect sizes were calculated as partial eta squared (*np2*). When significant main or interaction effects were observed, pairwise comparisons were performed using the Bonferroni post-hoc test. Sleep parameters, SI and Hooper’s sleep, stress, and muscle soreness scores being not normally distributed, were analyzed using Friedman nonparametric analysis of variance, and pairwise comparisons were conducted using the Wilcoxon test.

## RESULTS

### Objective and subjective sleep Parameters

Statistical analysis showed no significant difference during the night before experimental days in objective (i.e., TIB, TST, SE and WASO) and subjective (i.e., VAS) sleep parameters between the CON and NAP conditions.

### Heart rate

**FIG. 1:**
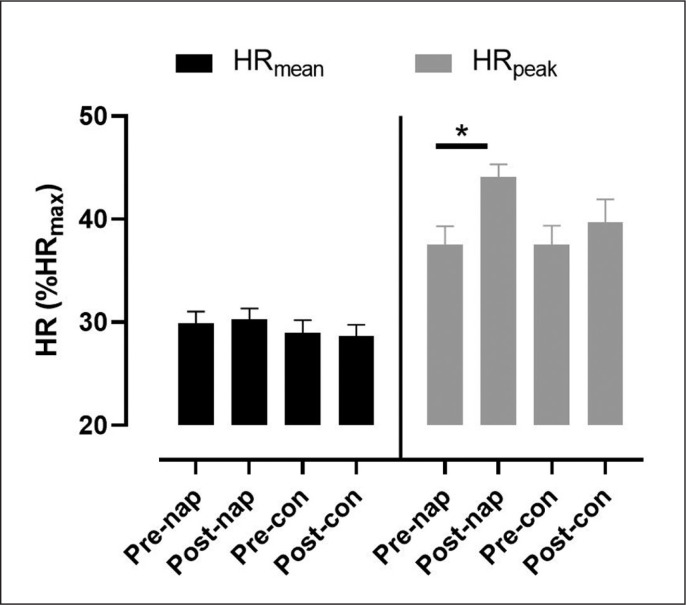
Mean values (± SEM) for HR_mean_ and HR_peak_ before and after NAP and CON conditions. *: significant difference (p < 0.05). Abbreviations: CON, control condition; NAP, nap condition; Pre, before nap/rest period; Post, after nap/rest period.

There was no significant effect of condition (F_(1,11)_ = 2.7, p > 0.05, *ŋp*^2^ = 0.19) or time (F_(1,11)_ = 0.002, p > 0.05, *ŋp*^2^ = 0.005) on HR_mean_. Two-way repeated measures ANOVA showed a significant effect of time (F_(1,11)_ = 6.1, p = 0.03, *ŋp*^2^ = 0.35) on HR_peak_. Bonferroni post-hoc test revealed that HR_peak_ was significantly higher in post-nap wakefulness compared to pre-nap wakefulness (p = 0.004) (Figure 1).

### HRV analysis

#### Frequency domain

**FIG. 2:**
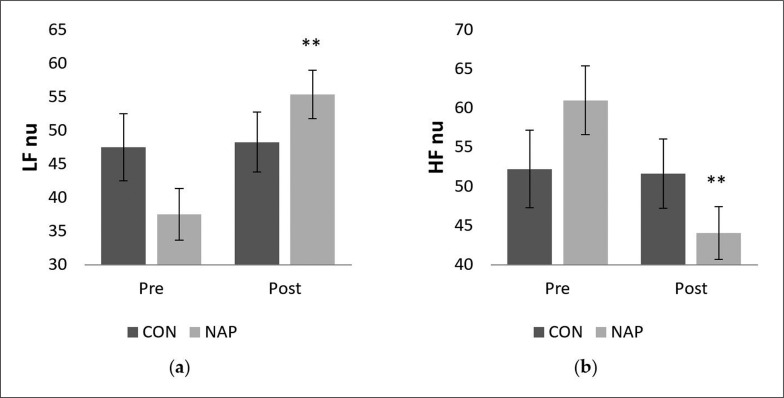
Mean values (± SEM) for (a) LF nu, and (b) HF nu before and after nap/rest. ** p < 0.01 significant difference compared to pre nap/rest. Abbreviations: CON, control condition; HF, high frequency; LF, low frequency; NAP, nap condition; nu, normalized unit; Pre, before nap/rest period; Post, after nap/rest period.

Statistical results revealed a significant interaction (condition × time) (F_(1,11)_ = 9.42, p = 0.01, *ŋp*^2^ = 0.46) on the normalized unit of LF (LF nu). There was no significant effect of time (F_(1,11)_ = 3.22, p > 0.05, *ŋp*^2^ = 0.22) or condition (F_(1,11)_ = 0.09, p > 0.05, *ŋp*^2^ = 0.008). Bonferroni post-hoc showed that LF nu was significantly higher after nap compared to before nap (P = 0.005) (Figure 2.a). Similarly, while no significant effect of time (F_(1,11)_ = 3.92, p > 0.05, *ŋp*^2^ = 0.26) and condition (F_(1,11)_ = 0.01, p > 0.05, *ŋp*^2^ = 0.001) was observed on the normalized unit of HF (HF nu), there was a significant interaction (condition × time) (F_(1,11)_ = 4.91, p = 0.04, *ŋp*^2^ = 0.30). HF nu was significantly lower after nap compared to before nap (p = 0.005) (Figure 2.b).

### Time-domain

**FIG. 3:**
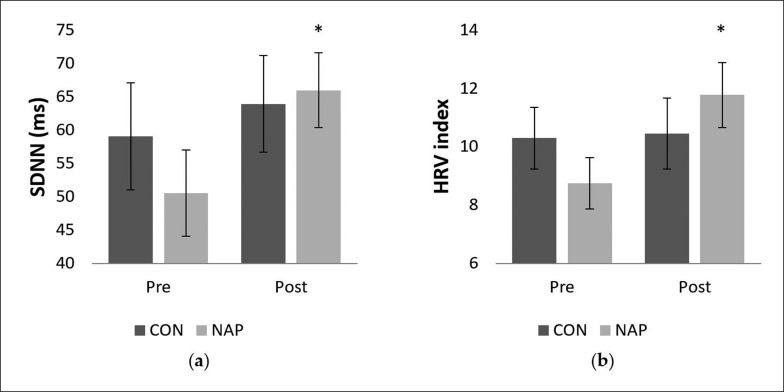
Mean values (± SEM) for (a) SDNN, and (b) HRV index before and after nap/rest. * p < 0.05 significant difference compared to pre nap/rest. Abbreviations: CON, control condition; NAP, nap condition; Pre, before nap/rest period; Post, after nap/rest period.

Concerning SDNN, repeated measure ANOVA tests revealed a significant effect of time (F_(1,11)_ = 5.03, p = 0.04, *ŋp*^2^ = 0.31). There was no significant effect of condition (F_(1,11)_ = 0.46, p > 0.05, *ŋp*^2^ = 0.04) or interaction (F_(1,11)_ = 1.04, p > 0.05, *ŋp*^2^ = 0.08). SDNN values increased significantly in post-nap wakefulness (p = 0.04 (Figure 3.a).

Regarding geometric analysis, the two-way repeated measures ANOVA revealed a significant interaction (condition × time) (F_(1,11)_ = 5.55, p = 0.03, *ŋp*^2^ = 0.33) on the HRV index. No significant effect of condition (F_(1,11)_ = 0.01, p = 0.8, *ŋp*^2^ = 0.002) or time (F_(1,11)_ = 2.74, p = 0.12, *ŋp*^2^ = 0.20) was reported. Values were significantly higher after nap compared to before nap (p = 0.03). Interestingly, there were no significant changes for the CON condition (Figure 3.b).

### Stress index

**FIG. 4:**
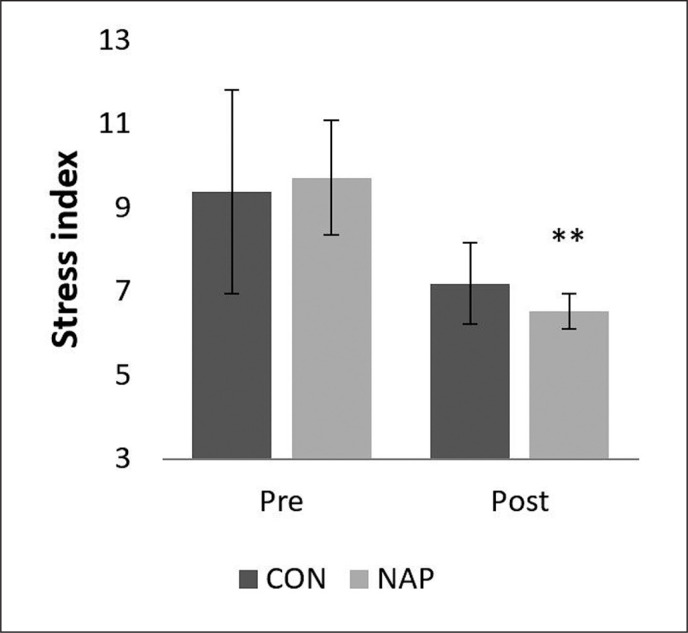
Mean values (± SEM) for Stress Index before and after NAP and CON conditions. ** p < 0.01 significant difference compared to pre nap/rest. Abbreviations: CON, control condition; NAP, nap condition; Pre, before nap/rest period; Post, after nap/ rest period.

A significant decrease was noticed in stress index (SI) during post-nap wakefulness compared to pre-nap wakefulness (p = 0.01). Results showed no significant changes in CON condition (Figure 4).

### ESS and Hooper scale

**FIG. 5:**
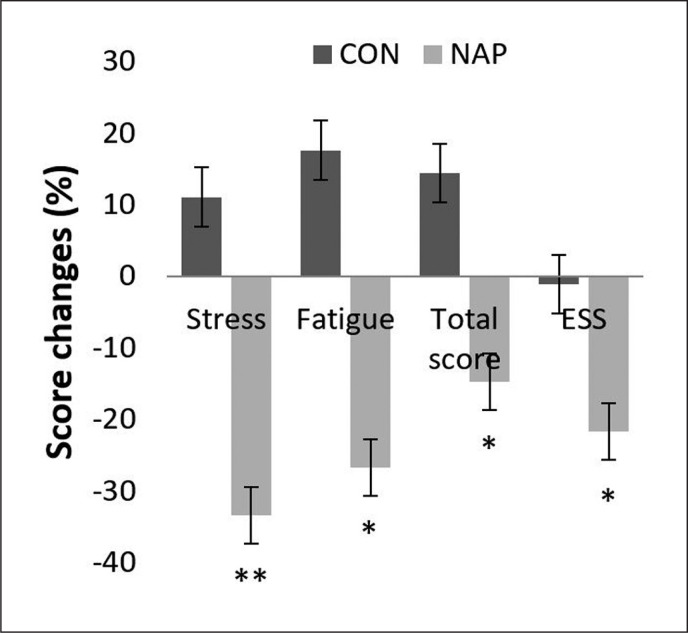
Percentage of score changes (± SEM) from pre to post nap/rest for Hooper’s stress, fatigue and total score, and ESS. * p < 0.05, ** p < 0.01 significant difference compared to pre nap/rest. Abbreviations: CON, control condition; ESS, Epworth sleepiness scale; NAP, nap condition; Pre, before nap/rest period; Post, after nap/rest period.

Repeated measure ANOVA tests showed a significant effect of time (F_(1,11)_ = 6.19, p = 0.03, *ŋp*^2^ = 0.36) and a significant interaction (condition × time) (F_(1,11)_ = 5.18, p = 0.04, *ŋp*^2^ = 0.32) on ESS. Bonferroni post-hoc test revealed that ESS was significantly lower after nap compared to before nap (p = 0.03). In the same way, a significant decrease was observed in Hooper’s post-nap stress, fatigue and total score compared to pre-nap (p = 0.009, p = 0.01, p = 0.04, respectively) (Figure 5).

## DISCUSSION

In this study, we aimed to investigate the impact of daytime sleep on cardiac autonomic activity (i) to fill the gap in the literature and (ii) to give insight regarding the beneficial effect of nap reported in our previous papers [7–10]. To our knowledge, this is the first study to investigate the effect of a 40-min daytime nap opportunity on HRV in elite basketball players. Our findings showed that the HRV profiles of participants changed after a daytime nap, while no significant changes were reported for the control condition. We noticed a significant increase in HR_peak_, SDNN, HRV index, and LF nu and a significant decrease in HF nu and SI following the nap opportunity. Moreover, daytime napping decreased Hooper’s stress, fatigue, total score, and subjective sleepiness according to ESS.

It is well established that changes in LF and HF reflect specific changes in cardiac autonomic regulation. There is wide consensus regarding the significance of the HF component, which reflects cardiac parasympathetic nerve activity [13, 24, 34]. In contrast, the LF band was assumed to reflect a dominant sympathetic effect [17, 34]. It is worth mentioning that the meaning of the LF component is still debated. Some researchers consider LF as a marker of sympathetic activity, while other investigators believe that the LF component of HRV is a reflection of fluctuations in both sympathetic and parasympathetic activity [13, 17, 34, 55]. The current study showed a significant increase in HR_peak_ and time-domain parameters of HRV (i.e., SDNN and HRV index) after NAP. In addition, we noticed a significant decrease in HF from pre- to post-nap wakefulness, accompanied by a simultaneous increase in LF. Interestingly, there were no significant changes in the CON condition. Taken together, these results suggest a sympathetic dominance following the nap opportunity.

Importantly, previous studies reported significant changes in HRV profile from wakefulness into sleep and across different sleep stages during nocturnal sleep [12, 13] as well as daytime naps [14, 15]. The limited number of studies investigating the effect of daytime napping on HRV using various experimental protocols reported a shift of the ANS from sympathetic to parasympathetic dominance from wakefulness into sleep [14, 15, 35, 36, 56]. This vagal dominance is characterized by a reduced heart rate coupled with increased HF activity and a marked reduction of LF bands. Throughout the progression of non-rapid eye movement (NREM) sleep, HF activity remains elevated, with higher vagal modulation compared to rapid eye movement (REM) sleep, suggesting an overall reduction in cardiovascular output and dominance of parasympathetic/vagal activity during NREM sleep [14, 15, 35, 36, 56]. Importantly, these fluctuations and dynamic changes in the autonomic profile are similar to those seen during nocturnal sleep [35], are associated with significant benefits for the cardiovascular system, and may be responsible for the homeostatic regulatory balance between sympathetic and vagal activity [12]. This balance has been correlated with reduced risk for cardiovascular disease, diabetes, and all-cause mortality [24], suggesting a cardioprotective function of sleep [12] which has led some researchers to describe normal sleep as a “cardiovascular holiday” [12].

Interestingly, only one study compared the impact of daytime napping on the ANS from pre- to post-nap wakefulness [56]. The study of AlQatari et al. [56] showed an increase in LF during post-nap wakefulness compared with that during pre-nap wakefulness. The current study showed significant changes regarding HRV profile from pre- to post-nap wakefulness, including a marked increase in LF. This result is in line with the previous report [56], indicating relative sympathetic dominance. Similar to the results of AlQatari et al. [56], we noticed a significant decrease in HF, which also supports the above-mentioned statement regarding the restoration of sympathetic dominance during post-nap wakefulness. Moreover, a significant increase in the LF/HF ratio has been measured after-compared to pre-nap wakefulness [56]. Although the LF/HF ratio was proposed as an index describing the balance between the two branches of the ANS, in which an increased ratio reflects sympathetic dominance and a reduction in this ratio indicates parasympathetic dominance [57], this index has not been taken into account in the present study due to the several limitations described in previous research [55]. According to Billman [55], the complex nature of LF power (i.e., the LF component of HRV is a reflection of fluctuations in both sympathetic and parasympathetic activity) and the non-linear interactions between sympathetic and parasympathetic nerve activity that are confounded by the mechanical effects of respiration and prevailing heart rate, make it impossible (i) to delineate the physiological basis for LF/HF with any degree of certainty and (ii) to quantify cardiac “sympathovagal balance”.

The current investigation revealed a significant decrease in SI during post-nap compared to pre-nap wakefulness. This result was confirmed by Hooper’s stress scores, which decreased significantly following the nap opportunity. In addition, the decrease in sleepiness and fatigue and the improvement of Hooper’s total score suggest a better readiness of athletes and could explain the enhancement of performance reported in previous studies with NAP [9, 10]. It is important to mention that these studies included sedentary, inactive participants (i.e., young [14, 15, 35, 36, 56] or older adults [36]). One study compared daytime nap to nocturnal sleep [35]. Another study aimed to investigate changes in HRV during daytime naps in young and older adults [36]. Unlike the abovementioned studies, the present investigation aimed to evaluate the effect of nap on ANS regulation in an elite athlete population. Furthermore, it is noteworthy to mention that moderation of sympathetic activity – indicated by greater high-frequency HRV values – has been reported to be associated with improved basketball shooting, passing and dribbling performance in previous research [58].

### Limitations

The present study presents some limitations. First, sleep was monitored using actigraphy, whereas, polysomnography provides more accurate results both in terms of quality and quantity and provides details regarding sleep stages [59]. Such information could have given us accurate information on the different changes in HRV profiles across sleep stages. Second, although this study was conducted on professional basketball players, only one basketball team was used, which could also lead to a lack of generalizability. Third, the selected participants were nonhabitual nappers. Given that non-habitual nappers display heavier sleep inertia at the awakening compared to habitual nappers [60], results could be different if habitual nappers were included instead of non-habitual nappers, especially regarding HRV indexes.

## CONCLUSIONS

The results of the present study showed that a 40 min nap opportunity increased global heart rate variability, operationalized through an increase in LF nu and a decrease in HF nu. In addition, daytime napping might be a successful strategy for reducing stress since (i) the stress index decreased significantly during post-nap wakefulness and (ii) according to the Hooper scale, subjective stress scores were lower following nap. Overall, these findings suggest that napping reduces stress, sleepiness, and fatigue, and might provide an advantage by preparing the body for a much-required sympathetic comeback following peaceful rest and parasympathetic dominance during sleep. Our results support the notion that daytime napping is beneficial for cardiovascular health in basketball professional athletes.

## Conflicts of Interest

The authors declare no conflict of interest.
